# A dual-branch selective attention capsule network for classifying kiwifruit soft rot with hyperspectral images

**DOI:** 10.1038/s41598-024-61425-4

**Published:** 2024-05-09

**Authors:** Zhiqiang Guo, Yingfang Ni, Hongsheng Gao, Gang Ding, Yunliu Zeng

**Affiliations:** 1 School of Information Engineering, Wuhan University of Technology, Wuhan, 430070 Hubei China; 2 National Key Laboratory for Germplasm Innovation & Utilization of Horticultural Crops, National R&D Centre for Citrus Preservation, Huazhong Agricultural University, Wuhan, People’s Republic of China

**Keywords:** Kiwifruit, Soft rot classification, Hyperspectral image classification, Attention mechanism, Capsule network, Computational models, Image processing, Machine learning, Optical spectroscopy, Plant sciences

## Abstract

Kiwifruit soft rot is highly contagious and causes serious economic loss. Therefore, early detection and elimination of soft rot are important for postharvest treatment and storage of kiwifruit. This study aims to accurately detect kiwifruit soft rot based on hyperspectral images by using a deep learning approach for image classification. A dual-branch selective attention capsule network (DBSACaps) was proposed to improve the classification accuracy. The network uses two branches to separately extract the spectral and spatial features so as to reduce their mutual interference, followed by fusion of the two features through the attention mechanism. Capsule network was used instead of convolutional neural networks to extract the features and complete the classification. Compared with existing methods, the proposed method exhibited the best classification performance on the kiwifruit soft rot dataset, with an overall accuracy of 97.08% and a 97.83% accuracy for soft rot. Our results confirm that potential soft rot of kiwifruit can be detected using hyperspectral images, which may contribute to the construction of smart agriculture.

## Introduction

Kiwifruit is popular among consumers for its tender flesh, sweet and sour taste, and high rich nutritional value. Therefore, the planting area of kiwifruit is constantly expanding in recent years. However, with continuously increasing kiwifruit production, various kiwifruit diseases are also emerging^1–5^, among which soft rot, a fungal rot disease of kiwifruit, causes the most serious postharvest loss. Early detection of soft rot is very important for kiwifruit growers, sellers, and researchers. Early and accurate detection of diseases can help to distinguish healthy fruit from diseased fruit, and prevent postharvest losses due to infection of healthy fruit^6^.

The diseased kiwifruit of soft rot generally shows no evident appearance characteristics at the early stage, and rapidly becomes soft at the later stage of disease. Typically, the diseased fruit shows round or oval spots with a watery ring around on the peel, and the flesh under the peel at the spot is creamy white, which can cause rot of the whole fruit in severe cases^7^. Because the symptoms are not obvious at the early stage of disease, manual or machine vision has limited effectiveness in distinguishing healthy kiwifruit from diseased ones^8^. In recent years, non-destructive detection techniques based on hyperspectral imaging (HSI) have gradually emerged. HSI technology can simultaneously obtain the image and spectral information of fruit, in which the image information can reveal the external shape characteristics, defects, and disease spots, while the spectral information can indicate various chemical components of the fruit, and at specific wavelengths, the images can more sensitively reflect the defects^9^. At present, HSI technology has been widely used in non-destructive detection of fruit quality and diseases^10–12^. For example, the study^11^ used HSI to detect fungal-infected strawberries. Sun et al.^13,14^ employed HSI to classify three common diseases of peaches. Pan et al.^15^ achieved a 97.5% accuracy using hyperspectral images combined with support vector machines in detecting black spot disease of pears. These studies have indicated the potential of using HSI to detect fruit diseases. However, most fruit disease classifications are conducted based on classical machine learning methods, which are somewhat blind in terms of feature extraction and classifier selection, and are lack of uniform selection criteria for different objects. In recent years, deep learning has been widely used in image classification due to its powerful representation and automatic learning of features^16–18^, and hyperspectral images are highly appropriate for deep learning research due to their rich information. For instance, Wang et al.^19^ used deep learning methods to improve the accuracy of detecting mechanical damage inside blueberries while reducing the computation time. Gao et al.^20^ employed convolutional neural networks to classify early and mature blueberries with a test set accuracy of 98.6%.The combination of hyperspectral data and deep learning has also been extensively studied in the field of remote sensing. Ma et al.^21^ proposed a dual-branch multi-attention mechanism network (DBMA) for HSI classification to separately extract the spectral and spatial features to reduce the mutual interference between them. Roy et al.^22^ proposed a hybrid spectral convolutional neural network (HybridSN) to reduce the complexity of the model and the risk of overfitting. Zhang et al.^23^ constructed the SPRN network for hyperspectral image classification by using residual learning and a spectral partitioning strategy.

Significant progress has been achieved in integrating deep learning with hyperspectral images, yet current research still faces limitations. For example, while some researchers have utilized CNN to automatically extract features, they simply reshape one-dimensional vectors of spectral information into two-dimensional pseudo-images for processing^24,25^; Others have trained the multiband images of hyperspectral images analogous to the 3-channel images of RGB images directly using basic convolutional neural networks such as AlexNet networks^20^. However most of these methods do not fully leverage the unique properties of hyperspectral images, particularly the integration of spatial and spectral information. Additionally, they often neglect network optimization specifically designed for fruit hyperspectral images. Hence, there is still potential to improve classification accuracy.

To fully extract the features of kiwifruit hyperspectral images, this work proposes a dual-branch selective attention capsule network (DBSACaps) for classification of kiwifruit soft rot. The main contributions are as follows. Firstly, feature extraction is divided into the spectral branch and spatial branch to separately extract the features so as to prevent their mutual interference. Secondly, a selective attention module is set to fuse spectral features and spatial features, which can improve the contribution of useful features to the classification. Finally, a capsule network instead of the convolutional neural network is used to obtain more robust features. Compared with other methods, the proposed network can achieve the highest classification accuracy.

## Materials and methods

### Dataset construction

#### Sample preparation

The ‘Yunhai No.1’ (Actinidia chinensis) were harvested from Chuyang orchard in Wuhan, Hubei Province, China. Fruit with ripeness at commercial harvest with soluble solids content of 8%–9%, and fruit weights of 80–110 g were selected as samples. In addition, after removal of deformed and scarred fruit, the remaining samples were treated with prochloraz and imazalil, and stored under cold storage as described in Asiche et al.^26^. Diaporthe ereswas used to inoculate samples at the central equator of the fruit. The experiment was divided into a healthy control group and an experimental group. The healthy control group was injected with sterile saline to eliminate the impact of the inoculated wound on classification. The experimental group was subjected to two doses (6 mL and 10 mL) of inoculation to simulate the onset of kiwifruit soft rot under different conditions. After inoculation, all fruit were single-bagged and stored with the inoculated side up at 20 °C. The day of inoculation was recorded as day 0, and hyperspectral images were acquired at 1:00 p.m. daily from day 1, while the development of soft rot was monitored until it became severe or white mycelium appeared.

#### Hyperspectral image acquisition and processing

A portable hyperspectral imager developed by Interuniversity Microelectronics Centre(IMEC), and Belgium was used for hyperspectral data acquisition^27^. The imager has 150 available spectral bands at 470–900 nm, with an imaging resolution of $800 \times 800$ pixels according to the size of kiwifruit. The camera integration time is 4 ms, and the analog gain is 1.6 dB. During image acquisition, the whole system was placed in a closed black box to prevent interference from external light sources.

**Figure 1:**
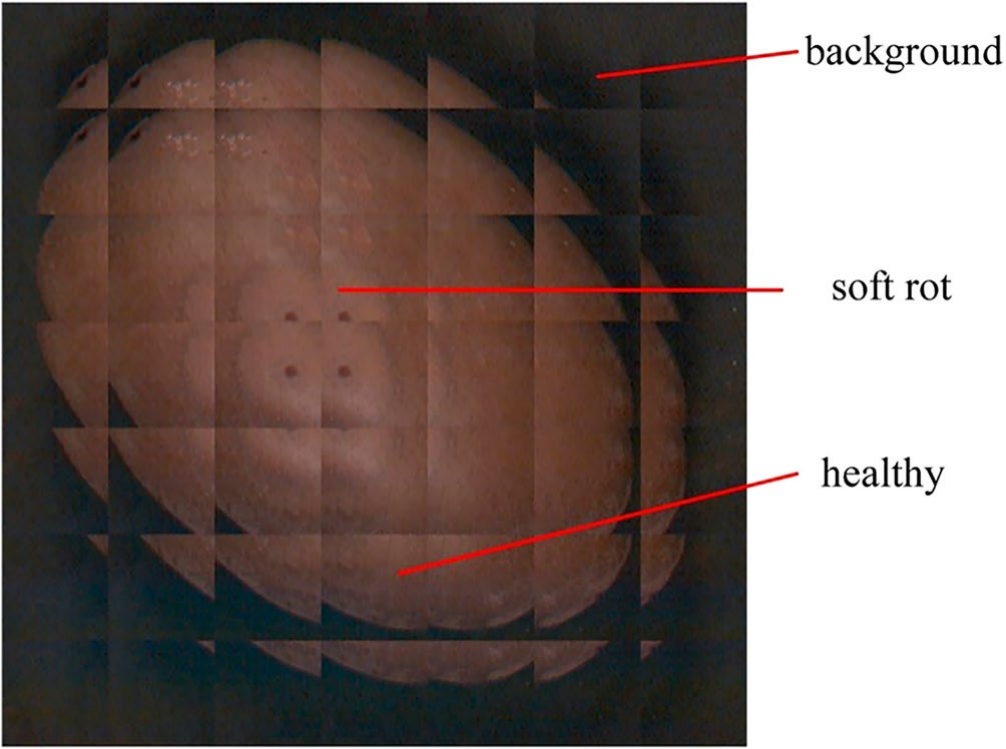
Blocking process of kiwifruit image. First, the image was normalized to a uniform image size of $256 \times 256$, and then divided into blocks of $64 \times 64$ with a step size of 32. the blocks were labeled as soft rot, healthy and background based on the percentage of soft rot. The figure highlights canonical examples representing each of the three categories.

Considering that soft rot occupies a relatively small portion of the whole image, a cut method similar to that used in remote sensing image classification was employed to segment the kiwifruit image into blocks as shown in Fig. 1. First, the image was normalized to a uniform image size of $256 \times 256$, and then divided into blocks of $64 \times 64$ with a step size of 32.

The labels for image blocks were made as follows: (1) when the percentage of soft rot exceeded 10%, the block was labeled as soft rot; (2) when the percentage of background pixels exceeded 50%, the block was labeled as background; (3) the remaining blocks were taken as healthy kiwifruit.

Firstly, the hyperspectral images before segmentation were divided into the training set, validation set, and test set at a ratio of 2: 1: 1, which were then respectively divided into blocks. Finally, the training set comprised a total of 1876 samples; the validation set 899 samples; and the test set 928 samples.

To obtain more images and improve the diversity of the dataset, data augmentation was carried out based on these block images by mirroring, random cropping, and adding shadow noise^28^. Since the background image features were relatively simple, only healthy images and soft rot images were augmented. After augmentation, a total of 11,194 samples were obtained. The specific number of samples is presented in Table 1.

**Table 1:** Dataset of kiwifruit soft rot disease.

Data augmentation	Category	Training set	Validation set	Test set	Sample size
Before augmentation	Background	600	293	313	1206
	Healthy	630	334	304	1268
	Soft rot	646	272	311	1229
After augmentation	Background	600	293	313	1206
	Healthy	2520	1336	1216	5072
	Soft rot	2584	1088	1244	4916

The specific number of samples for each category before and after dataset augmentation is presented in Table.

### Model architecture

RGB image is a cube with two spatial dimensions and one channel dimension, and has three channels of red, green, and blue in the channel dimension. Comparatively, hyperspectral images have significantly more channels than RGB images. The data of hyperspectral images are much larger at the same spatial resolution, and the RGB image classification network cannot capture the relationship between spectra. In addition, another problem of hyperspectral images is the small data sets. Unlike large RGB image data sets that can be used for pre-training, hyperspectral images do not have large data sets to be used for pre-training, which also limits the depth and width of the network due to limited training samples.

For remote sensing image classification networks, single-branch networks, such as FDSSC^19^, SSRN^29^, and HybridSN^22^, extract spectral features and spatial features successively. Since the two kinds of features are in different domains, when extracting spatial features, the spectral features are easily destroyed. Different from single-branch networks, the DBMA^21^ network divides the feature extraction into spectral and spatial branches to separately extract the corresponding features, which reduces the mutual interference between the two types of features. However, the large depth of feature extraction of the network leads to longer training time, and there is a lack of fusion mechanism in the direct superposition of spectral and spatial information, which negatively affects the classification performance.

**Figure 2:**
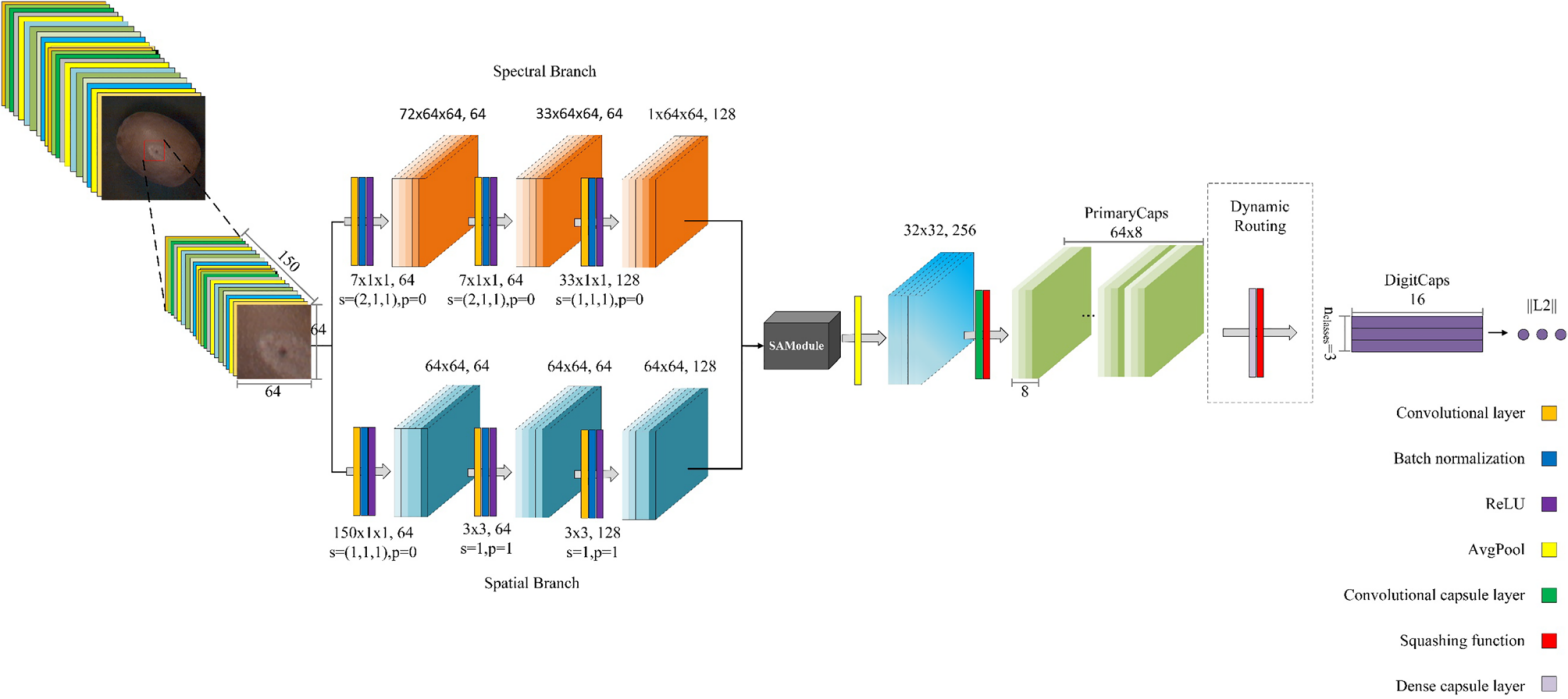
DBSACaps network structure. Figure 2 illustrates the overall structure of the network, which consists of three parts. The first part is feature extraction, which is divided into spectral branch and spatial branch for feature extraction; the second part is feature fusion, which uses the attention mechanism (The SAModule module is shown in Fig. 3) to select features from different branches; and the third part is the classification by the capsule network. The operation process in the network is represented by corresponding color blocks, and the corresponding relationship is shown in the legend in the bottom right corner of the figure.

To address the above problems, we propose a hyperspectral image classification network based on a dual-branch selective attention and capsule network for kiwifruit soft rot classification. Figure 2 illustrates the overall structure of the network, which consists of three parts. The first part is feature extraction, which is divided into spectral branch and spatial branch for feature extraction; the second part is feature fusion, which uses the attention mechanism to select features from different branches; and the third part is the classification by the capsule network. Next, we will introduce these three parts in detail.

#### Dual branch feature extraction

The proposed network is an end-to-end network without relying on feature engineering for dimensionality reduction. For a hyperspectral image cube of size $C \times H \times W$, it can be directly fed into the network to use CNN for dimensionality reduction and feature extraction. For example, when using 3DCNN to extract features, a convolutional kernel of $7 \times 3 \times 3$ can learn both spectral and spatial features. To enable feature separation, we use a dual-branch structure with kernels of different sizes to separately extract spectral and spatial features.

For the spectral branch, a 3DCNN with a convolution kernel size of $d \times 1 \times 1$ is used. The two-dimensional convolution process aims to extract local spatial features, while a 3DCNNN of $d \times 1 \times 1$ does not extract any spatial elements as it does not consider the relationship between pixel points in a spatial image and their neighboring pixels, and convolution of $1 \times 1$ size allows a linear combination of each pixel in a spatial image. Therefore, for hyperspectral images, using a 3DCNN of $d \times 1 \times 1$ size can extract spectral features and well preserve spatial features.

For hyperspectral image data, the dimensionality can be reduced by using 3DCNN and reshaping. The key to dimensionality reduction lies in the padding method of 3DCNN. Same padding and valid padding are two commonly used padding methods. Same padding means that before convolution, the input feature is subjected to zero-padding, and the convolution results at the boundary can be preserved. Usually, the output shape is the same as the input shape. Valid padding means that the boundary data are not processed, and $d \times 1 \times 1$ size of 3DCNN combined with valid padding can achieve dimensionality reduction and retain the original spatial information.

For the spatial branch, after using 3DCNN to reduce the dimensionality and reshape it into a 2D image, the use of a 2DCNN with a convolution kernel size of $A \times A$ can effectively extract spatial features. The details of feature extraction are shown in Table 2.

**Table 2:** Feature extraction network structure and parameters.

Branch	Layer	Kernel size	Stride	Padding	Activation	Output size
/	Input	/	/	/	/	1150 × 64 × 64
Spectral	3DConv_1	7 × 1 × 1	(2,1,1)	0	ReLU	64,72 × 64 × 64
	3DConv_2	7 × 1 × 1	(2,1,1)	0	ReLU	64,33 × 64 × 64
	3DConv_3	33 × 1 × 1	1	0	ReLU	128,1 × 64 × 64
	Reshape_1	/	/	/	/	128,64 × 64
Spatial	3DConv_4	150 × 1 × 1	1	0	ReLU	64,1 × 64 × 64
	Reshape_2	/	/	/	/	64,64 × 64
	2DConv_1	3 × 3	1	1	ReLU	64,64 × 64
	2DConv_2	3 × 3	1	1	ReLU	128,64 × 64

The network structure and parameters of Dual Branch Feature Extraction (including convolution kernel size, step size, padding, activation function, output size) are presented in the Table.

#### Feature fusion

**Figure 3:**
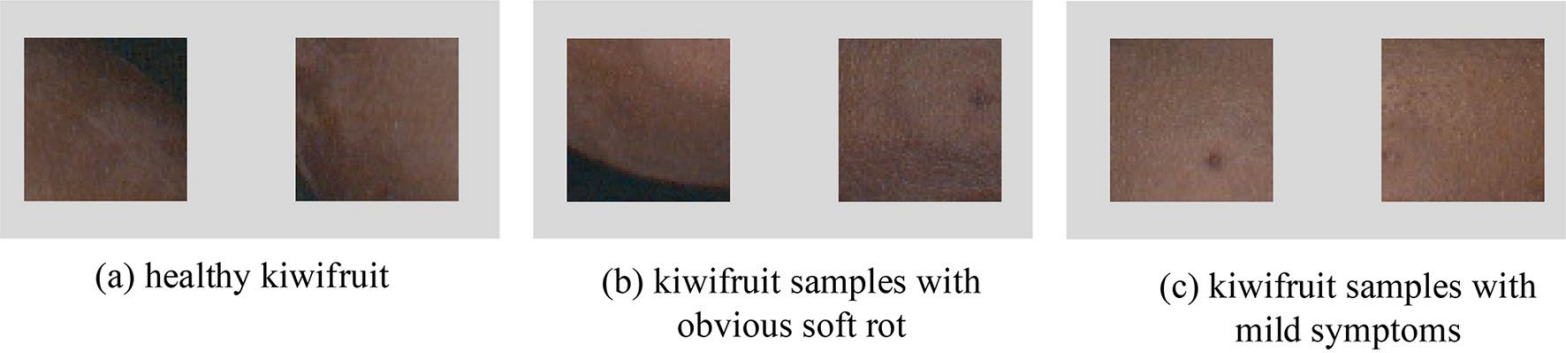
Partial image of the kiwifruit dataset. Figure 3 shows a schematic diagram of some samples. It can be seen that for kiwifruit samples with obvious soft rot, their spatial characteristics are significantly different from those of healthy kiwifruit. However, for kiwifruit samples with mild symptoms, the feature differences are small on the spatial image. For these samples, spectral features play a more important role in the classification.

After dual-branch feature extraction, the spectral features and spatial features can be fully extracted without mutual interference. However, for current common dual-branch classification networks, most spectral and spatial features are directly aggregated in the feature dimension for classification. For the kiwifruit soft rot data set used in this study, Fig. 3 shows a schematic diagram of some samples. It can be seen that for kiwifruit samples with obvious soft rot, their spatial characteristics are significantly different from those of healthy kiwifruit. However, for kiwifruit samples with mild symptoms, the feature differences are small on the spatial image. For these samples, spectral features play a more important role in the classification.

**Figure 4:**
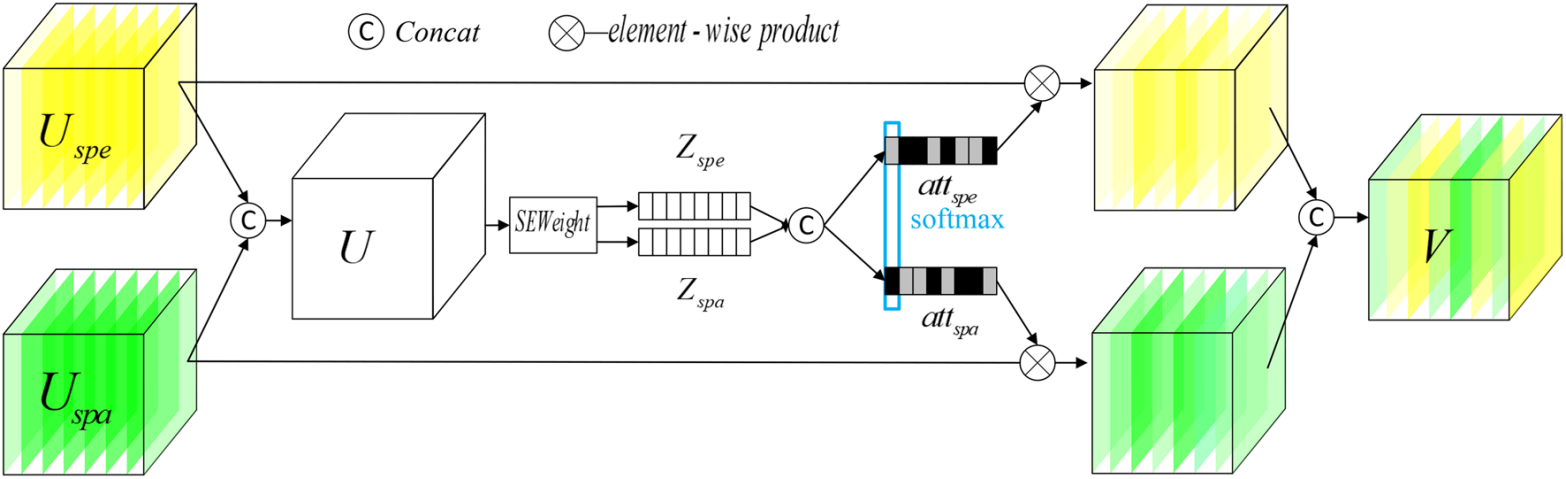
Selective attention module. The two input branches are spectral features $U_{spe}$ and spatial features $U_{spa}$ extracted by the feature extraction module. The main process of Concat is to superimpose the spectral features and spatial features in the channel dimension to obtain a fusion feature map, and then use $SEWeight$^31^ to extract the attention weight information to obtain the attention weight vectors of different branches. $U$,$Z_{spe}$ and $Z_{spa}$ are respectively defined by Eqs. (1) and (2). For the Select part, the main purpose is to fuse the attention weight vectors of the two branches, and then recalibrate the fused vector. We chose the Softmax activation function to complete it. $att_{spe} ,att_{spa}$ are respectively defined by Eq. (4). Finally, the attention vector and the feature map are multiplied along the channel dimension to obtain the attention feature map, and the attention feature maps of the two branches are superimposed along the channel dimension to obtain the fusion feature. The formula is as follows, in which $\otimes$ means the multiplication of corresponding elements along the channel dimension. $V$ is defined by Eq. (5).

If the dual-branch features are aggregated directly, it is equivalent to giving the same weight to the spatial and spectral features, which is unfavorable for the classification of some samples. Inspired by SKNet^30^, we designed a selective attention module (SAModule), which assigns different weights to spatial features and spectral features before feature fusion, improving the classification accuracy of hyperspectral images. The selective attention module is shown in Fig. 4.

The module mainly includes two parts: Concat and Select. The two input branches are spectral features $U_{spe}$ and spatial features $U_{spa}$ extracted by the feature extraction module. The main process of Concat is to superimpose the spectral features and spatial features in the channel dimension to obtain a fusion feature map, and then use $SEWeight$^31^ to extract the attention weight information to obtain the attention weight vectors of different branches.

1 $$U = concat(U_{spe} ,U_{spa} )$$

2 $$Z_{s} = SEWeight(U_{s} ),$$

where $U \in \Re^{C \times S \times S}$,$C$ is the number of channels after $concat$, and $S \times S$ is the space size of the feature map. $U_{s} \in \Re^{{\frac{C}{2} \times S \times S}}$ refers to $U_{spe}$ or $U_{spa}$, $Z_{s}$ refers to $Z_{spe}$ or $Z_{spa}$, which are attention weight vectors corresponding to spectral features or spatial features, respectively.

For the Select part, the main purpose is to fuse the attention weight vectors of the two branches, and then recalibrate the fused vector. We chose the Softmax activation function to complete it.

3 $$Z = concat(Z_{spe} ,Z_{spa} )$$

4 $$att_{spe} = \frac{{\exp (Z_{spe} )}}{{\exp (Z_{spe} ) + \exp (Z_{spa} )}},att_{spa} = \frac{{\exp (Z_{spa} )}}{{\exp (Z_{spe} ) + \exp (Z_{spa} )}} ,$$

where $att_{spe} ,att_{spa}$ correspond to the attention vector of $U_{spe} ,U_{spa}$, respectively.

Finally, the attention vector and the feature map are multiplied along the channel dimension to obtain the attention feature map, and the attention feature maps of the two branches are superimposed along the channel dimension to obtain the fusion feature. The formula is as follows, in which $\otimes$ means the multiplication of corresponding elements along the channel dimension.

5 $$V = concat((att_{spe} \otimes U_{spe} ),(att_{spa} \otimes U_{spa} )).$$

#### Capsule network classification

In a convolutional neural network, a single convolutional layer is used to extract local features, and it is easy to ignore the spatial location relationship between features. When low-level features need to be combined to extract deep complex features, it is necessary to use a pooling layer to reduce the size of the feature map, and the pooling operation will lead to the loss of some information such as location information, and the increase in pooling operation requires more training data to compensate for this lost information. To solve these problems, Sabour et al.^32^ proposed the Capsule Network (CapsNet). Capsule is defined as a group of neurons instead of a single neuron, and represents an entity by using vectors instead of scalars. The activation of neurons in the capsule represents various properties of the entity, such as position, direction, size, and texture, and the vector length is used to represent the probability for the existence of the entity.

After the capsule network was proposed, many researchers attempted to apply CapsNet for the classification of HSI and achieved high accuracy. Ma et al.^33^ a two-dimensional capsule network to extract features and then classify them after PCA. Paoletti et al.^34^ extended the two-dimensional capsule network to a three-dimensional one to extract the spectral and spatial features of hyperspectral images. Jiang et al.^35^ proposed a dual-channel capsule network, which uses two separate convolution channels to extract the spectral and spatial features, and then connects and sends them to the capsule layer for classification. These studies have demonstrated the potential of CapsNet in HSI classification tasks. Hence, after the fusion of spectral and spatial features, the capsule network is used instead of a convolutional neural network to further extract features. In DBSACaps, the number of output capsules in the primary capsule layer is 64 and the capsule vector length is 8. The number of output capsules in the digital capsule layer is 3, namely the number of categories, and the capsule vector length is 16. The existence probability of each category can be obtained by calculating the modulus length of the output capsule of the digital capsule layer. Since the network does not use a reconstruction network, the loss function used in this network only includes the margin loss function.

### Evaluation index and experimental setup

In our experiments, all experiments were performed on a workstation equipped with Inter Xeon CPU E5-2620 v4 @ 2.1 GHz, Nvidia Geforce RTX2080Ti GPU, 64G RAM. For a fair comparison, all classification networks were implemented using the Pytorch library and the Python language. The experimental parameters of the proposed DBSACaps network are: the number of capsule network routing is set to 3; the batch size is 16; the initial learning rate is 0.000005; the training epoch is 100; the Adam optimizer is used for optimization; and the weight decay is 0.000001. The main classification evaluation metrics include overall accuracy (OA), average accuracy (AA), precision, recall rate, and F1 score.

To demonstrate the effectiveness of the proposed method, it was compared with several other widely used methods as ResNet50^36^, CapsuleNet^34^, DBMA^21^, HybridSN^22^, HS-CNN^37^, LMFN^38^, SPRN^23^.

In the comparison networks, Adam optimizer is used to update the parameters, and the training epoch is fixed at 100. Considering the different convergence speeds of different networks, we tested four learning rates for different networks, including 0.001, 0.0001, 0.00001, and 0.000005, and used the learning rate with the best performance in the case of not overfitting.

### Research involving plants

The study complies with the IUCN Policy Statement on Research Involving Species at Risk of Extinction and the Convention on the Trade in Endangered Species of Wild Fauna and Flora. The kiwifruits were used in this study. ‘Yunhai No.1’ (*Actinidia chinensis*) were harvested from Chuyang orchard in Wuhan, Hubei Province, China.

## Results and discussion

### Classification results of different networks

The overall classification results of different networks on the test set are presented in Table 3, and those of each category are shown in Table 4. The data marked in bold indicate the highest classification accuracy for that category and the italicized data represent the second highest.

**Table 3:** Comparison of classification results of different networks.

Network	Learning rate	Average accuracy (%)	Overall accuracy (%)
ResNet50	0.00001	96.65	95.74
CapsuleNet	0.000005	96.80	*96.36*
DBMA	0.0001	96.02	95.67
HybridSN	0.00001	94.96	93.69
HS-CNN	0.0001	*96.90*	95.96
LMFN	0.001	96.43	96.00
SPRN	0.00001	95.57	94.30
HPDM-SPRN	0.00001	94.82	94.16
DBSACaps	0.000005	**97.64**	**97.08**

The overall classification results of different networks on the test set are presented in Table. The results shows that the proposed DBSACaps network has the best performance among all methods, with an OA and AA of 97.08% and 97.64%, respectively, which are 0.72% and 0.74% higher than those of the second highest network (CapsuleNet and HS-CNN, respectively).

The highest value in each column is bold and the second highest is italicized.

**Table 4:** Evaluation indexes of different network categories.

Network	Background			Healthy			Soft rot			Average precision	Average recall	Average F1
	Precision	Recall	F1	Precision	Recall	F1	Precision	Recall	F1			
ResNet50	0.9780	*0.9936*	*0.9857*	0.9463	0.9572	0.9518	0.9633	0.9486	0.9559	0.9625	0.9665	0.9644
CapsuleNet	**0.9968**	0.9808	**0.9887**	0.9435	*0.9753*	*0.9592*	*0.9760*	0.9477	*0.9617*	*0.9721*	0.9680	*0.9699*
DBMA	*0.9902*	0.9712	0.9806	0.9597	0.9408	0.9502	0.9458	*0.9686*	0.9571	0.9653	0.9602	0.9626
HybridSN	0.9656	0.9872	0.9763	0.9082	0.9523	0.9297	0.9601	0.9092	0.9339	0.9477	0.9496	0.9467
HS-CNN	0.9720	**0.9968**	0.9842	0.9313	**0.9803**	0.9551	**0.9872**	0.9301	0.9578	0.9635	*0.9690*	0.9657
LMFN	0.9714	0.9776	0.9745	*0.9601*	0.9498	0.9549	0.9570	0.9654	0.9612	0.9628	0.9643	0.9635
SPRN	0.9511	0.9936	0.9719	0.9239	0.9482	0.9359	0.9608	0.9252	0.9427	0.9452	0.9557	0.9501
HPDM-SPRN	0.9902	0.9681	0.9790	0.9257	0.9424	0.9340	0.9455	0.9341	0.9397	0.9538	0.9482	0.9509
DBSACaps	0.9780	*0.9936*	*0.9857*	**0.9765**	0.9572	**0.9668**	0.9636	**0.9783**	**0.9709**	**0.9727**	**0.9764**	**0.9745**

The highest value in each column is bold and the second highest is italicized.

The each category classification results of different networks on the test set are presented in Table. As shown in Table 4, different networks have different effects on image classification for the three categories. In all the three metrics, our proposed DBSACaps network has the highest average precision rate, average recall rate, and average F1 index.

Table 3 shows that the proposed DBSACaps network has the best performance among all methods, with an OA and AA of 97.08% and 97.64%, respectively, which are 0.72% and 0.74% higher than those of the second highest network (CapsuleNet and HS-CNN, respectively). Besides the proposed network, CapsuleNet and HS-CNN are the two networks with relatively better performance, which may be ascribed to the following reasons. First, the CapsuleNet network can extract more features from the kiwifruit dataset due to its outstanding ability to capture the correlation of sample features, which contributes to its better classification performance compared with that of other networks. Secondly, the HS-CNN network is specifically proposed for avocado and kiwifruit ripeness, and therefore has a relatively good generalization performance with the dataset of this study.

Among the four networks of DBMA, HybridSN, LMFN, and SPRN, the LMFN network seems to have the best performance, which may be due to the sample size and characteristics of the data set in this study. The performance of HybridSN and SPRN is not comparable to that of DBMA, because both of them are single-branch networks, in which spectral features and spatial features easily interfere with each other.

The spatial attention module HPDM proposed in SPRN was proved to improve the classification accuracy of the network; however, the use of this module decreased the classification accuracy for the data set in this study, which can be mainly attributed to the difference between the two data sets. For the public dataset used in SPRN, although the block algorithm is used, the label of each patch only depends on the central pixel, while all other pixels have their own labels. The HPDM module is designed to prevent the degradation of classification accuracy caused by the interference of other pixels with increasing patch size. In this work, there is only one label for each patch. Hence, other pixels outside the center pixel can well be used for classification without interference, which may explain the decrease in accuracy after using the HPDM module.

As shown in Table 4, different networks have different effects on image classification for the three categories. In all the three metrics, our proposed DBSACaps network has the highest average precision rate, average recall rate, and average F1 index. In the background images, the CapsuleNet network has the best classification effect, with the highest precision rate and F1 index, and the DBSACaps network has the second highest recall rate and F1 index. Obviously, all networks have better classification results on the background images, probably because the background images have simpler features and have not been expanded with data. In healthy images, the DBSACaps network showed the highest precision rate and F1 index, and in soft rot images, this network had the highest recall rate and F1 index, which is consistent with the purpose of kiwifruit soft rot classification. The high precision rate of healthy images indicates a low probability of misreporting soft rot images as healthy by the network, and the high recall rate of soft rot images indicates that the network can well detect the potential soft rot disease.

### Effectiveness of dual branch

In order to validate the superiority of dual-branch feature extraction, three comparative network models were designed, namely HybridCaps, SpectralCaps, and SpatialCaps. Among them, SpectralCaps and SpatialCaps only use the spectral and spatial branches to extract spectral and spatial features in the feature extraction module, respectively; HybridCaps uses a single-branch feature extraction structure. Specifically, three consecutive 3DCNN is used to extract the spectral features, and then 2DCNN is used to extract spatial features after reshaping. None of the three networks requires a feature fusion module, and thus the capsule network is directly used for classification after feature extraction. The experimental results are shown in Table 5.

**Table 5:** Experimental results of dual-branch effectiveness.

Network	Background	Healthy	Soft rot	Average accuracy (%)	Overall accuracy (%)
HybridCaps	99.04	**97.04**	95.50	97.19	96.57
SpectralCaps	**99.36**	95.64	96.86	97.29	96.61
SpatialCaps	98.08	95.97	95.66	96.57	96.07
DBSACaps	**99.36**	95.72	**97.83**	**97.64**	**97.08**

Table shows that DBSACaps has higher OA and AA than HybridCaps, indicating that compared with the extraction of hybrid features with a single branch, extraction of features with dual branches is more favorable for the classification of kiwifruit soft rot.

The highest value in each column is in bold.

Table 5 shows that DBSACaps has higher OA and AA than HybridCaps, indicating that compared with the extraction of hybrid features with a single branch, extraction of features with dual branches is more favorable for the classification of kiwifruit soft rot. In addition, SpectralCaps is more powerful in predicting the category of soft rot, while SpatialCaps performs better in predicting the category of health, indicating that spectral features may be more important in distinguishing soft rot kiwifruit. Extraction of hybrid features with a single branch can improve the classification accuracy of the health category, but decrease that of soft rot, further indicating that single-branch extracted hybrid features tend to weaken the spectral features due to mutual interference between spatial and spectral features.

### Effectiveness of selective attention and capsule networks

To validate the effectiveness of the selective attention module on feature fusion and capsule network classification, three comparative network models were designed, namely DBNet, DBSA, and DBCaps. DBNet uses a layer of 2DCNN and then a fully connected layer to replace the capsule network for classification after dual-branch feature extraction. DBSA employs SAModule for feature fusion based on DBNet, and DBCaps does not use SAModule relative to DBSACaps. The DBNet and DBSA networks use fully connected layers for classification, and thus the loss function uses cross-entropy loss, and DBCaps uses the margin loss function. The experimental results are shown in Table 6.

**Table 6:** Experimental results of selective attention and capsule network effectiveness.

Network	Background	Healthy	Soft rot	Average accuracy (%)	Overall accuracy (%)
DBNet	99.36	95.89	92.93	96.06	94.95
DBSA	**99.68**	**99.10**	90.43	96.40	95.28
DBCaps	99.36	96.88	95.90	97.38	96.72
DBSACaps	99.36	95.72	**97.83**	**97.64**	**97.08**

Table shows that DBNet is the benchmark network with the fewest modules, with an OA of 94.95% and an AA of 96.06%. Compared with DBNet, the DBSA network has a 0.33% increase in OA and a 0.34% increase in AA; compared with DBCaps, the DBSACaps network has a 0.36% increase in OA and a 0.26% increase in AA, and the use of SAModule improves the classification accuracy of soft rot images by 1.93%. These results demonstrate the effectiveness of SAModule for dual-branch feature fusion.

The highest value in each column is in bold.

Table 6 shows that DBNet is the benchmark network with the fewest modules, with an OA of 94.95% and an AA of 96.06%. Compared with DBNet, the DBSA network has a 0.33% increase in OA and a 0.34% increase in AA; compared with DBCaps, the DBSACaps network has a 0.36% increase in OA and a 0.26% increase in AA, and the use of SAModule improves the classification accuracy of soft rot images by 1.93%. These results demonstrate the effectiveness of SAModule for dual-branch feature fusion.

**Figure 5:**
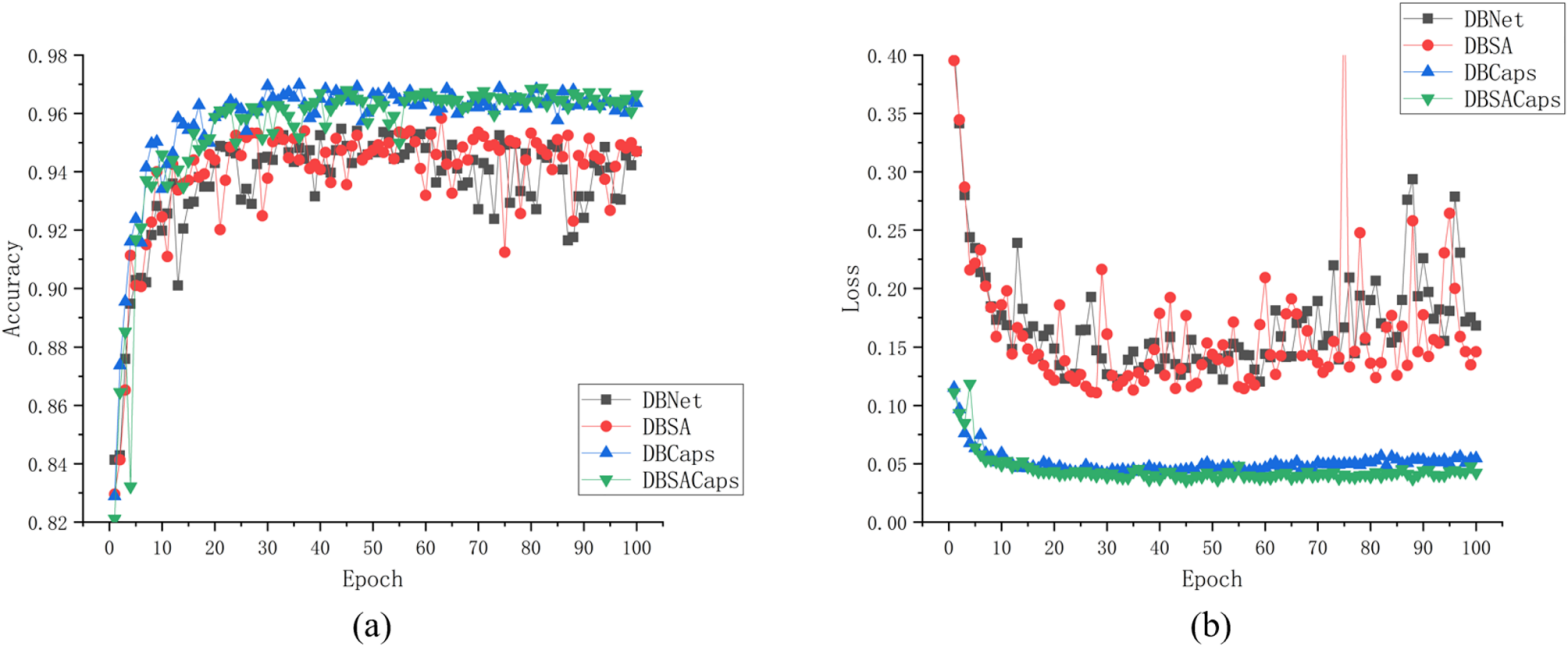
Validation set accuracy and loss curve. (**a**) shows the acc curves for the validation set of these four networks during training. (**b**) shows the acc curves for the validation set of these four networks during training. Different networks are distinguished by curves of different colors.

DBCaps improves the OA by 1.77% and AA by 1.32% compared to DBNet, and DBSACaps increases the OA by 1.8% and AA by 1.24% compared to DBSA. Figure 5 shows the acc and loss curves for the validation set of these four networks during training. It can be seen that the networks converge faster when using the capsule network for classification, and the loss is much smaller than that using the fully connected layer for classification, indicating that the capsule network has a significant advantage in the kiwifruit soft rot dataset.

### Experiments on reconstruction network

Capsule networks usually use reconstruction networks to further enhance model interpretability after the output of digital capsule layers. In order to explore whether the proposed DBSACaps network needs reconstruction networks, three comparison networks named as DBSACapsWithRec1, DBSACapsWithRec2, and DBSACapsWithRec3 were designed. DBSACapsWithRec1 uses the reconstruction network in the original capsule network, and employs three fully connected layers to complete the reconstruction of images. Since the image size used in this paper is $150 \times 64 \times 64$, the fully connected layer will cause a large number of parameters. Therefore, the DBSACapsWithRec2 network was constructed, in which images are first reconstructed at a $64 \times 64$ size in the fully connected layer, and then reconstruction of the spectral band dimension is completed using a layer of 2DCNN. The DBSACapsWithRec3 network employs the upsampling process in image segmentation^39^. In the reconstruction process of the fully connected layer, images are first reconstructed at a $32 \times 32$ size, and then restored to the size of $64 \times 64$ through transposed convolution, and finally reconstruction of the spectral band dimension is completed using a layer of 2DCNN. The experimental results are shown in Table 7.

**Table 7:** Experimental results of reconstruction network.

Network	Parameters	Background	Healthy	Soft rot	Average accuracy (%)	Overall accuracy (%)
DBSACapsWithRec1	125,793,752	**99.36**	96.38	95.34	97.03	96.25
DBSACapsWithRec2	8,005,380	**99.36**	95.07	96.86	97.10	96.36
DBSACapsWithRec3	7,422,574	**99.36**	**99.10**	94.13	97.53	96.90
DBSACaps	**7,135,312**	**99.36**	95.72	**97.83**	**97.64**	**97.08**

Table shows that the DBSACapsWithRec1 network directly uses the fully connected layer to reconstruct the image, and as a result, the number of parameters increases from 7 to 100 million. Under the limited training samples, the classification effect of the network is largely reduced. However, DBSACapsWithRec2, the number of network parameters drops significantly to 8 million, and there are also slight increases in OA and AA. In the DBSACapsWithRec3 network, the amount of parameters is further reduced, and the classification accuracy is close to that of DBSACaps. The OA and AA of the DBSACapsWithRec3 network do not exceed those of DBSACaps, and the use of the reconstruction network improves the accuracy of healthy images but reduces that of soft rot images. Therefore, the reconstruction network is not used in the proposed network.

The highest value in each column is in bold.

Table 7 shows that the DBSACapsWithRec1 network directly uses the fully connected layer to reconstruct the image, and as a result, the number of parameters increases from 7 to 100 million. Under the limited training samples, the classification effect of the network is largely reduced. However, DBSACapsWithRec2, the number of network parameters drops significantly to 8 million, and there are also slight increases in OA and AA. In the DBSACapsWithRec3 network, the amount of parameters is further reduced, and the classification accuracy is close to that of DBSACaps. The OA and AA of the DBSACapsWithRec3 network do not exceed those of DBSACaps, and the use of the reconstruction network improves the accuracy of healthy images but reduces that of soft rot images. Therefore, the reconstruction network is not used in the proposed network.

## Conclusion

In this study, hyperspectral imaging system and deep learning were combined for kiwifruit soft rot disease detection. And a dual-branch classification model based on attention and capsule networks is proposed according to the characteristics of the kiwifruit soft rot disease dataset. The proposed network avoids the mutual interference of the two types of features by separately extracting the spatial and spectral features using dual branches. In addition, in the feature fusion stage, the attention mechanism is used to redistribute the weights of different features, and in this way the network pays more attention to the features with greater contributions to the classification results. Finally, the use of capsule networks effectively reduces the training loss. Compared with other image classification networks, the DBSACaps network has better classification performance on the kiwifruit soft rot dataset. This study experimentally demonstrates the feasibility of using deep learning and hyperspectral images for soft rot classification of kiwifruit, providing additional support and possibilities for fruit disease detection. However, this data was based on a single cultivar, future work will involve collecting kiwifruit samples of various qualities from different regions for testing. This will help in adaptively adjusting model parameters to broaden the model’s applicability.

Additionally, the hyperspectral images in this study contain 150 bands, resulting in substantial data storage requirements. Future research will explore the selection of spectral bands to achieve higher accuracy with fewer bands. This approach will also consider how to maintain model accuracy with fewer samples, potentially aiding in the development of cost-effective multispectral cameras.

### Supplementary Information

Supplementary Information.

## Data Availability

The datasets used and analyzed during the current study are available from the corresponding author upon reasonable request.
