# Supplementary material for: A dual-branch selective attention capsule network for classifying kiwifruit soft rot with hyperspectral images
Source: Sci Rep. 2024 May 9;14:10664. doi: 10.1038/s41598-024-61425-4 (PMC11082204; doi:10.1038/s41598-024-61425-4)
Supplement: Supplementary file 1 — Supplementary Information. [file 41598_2024_61425_MOESM1_ESM.docx]

**Supplementary Table**

Table R1 Anova Test of different networks

|  | df | Sum_sq | Mean_sq | F | PR(>F) |
| --- | --- | --- | --- | --- | --- |
| Between Groups | 8 | 0.003731 | 0.000466 | 29.577419 | 1.328076*10-13 |
| Residual | 36 | 0.000568 | 0.000016 | / | / |

Table R2 Tukey HSD Test Results

|  | ResNet50 | CapsuleNet | DBMA | HybridSN | HS-CNN | LMFN | SPRN | HPDM-SPRN | DBSACaps |
| --- | --- | --- | --- | --- | --- | --- | --- | --- | --- |
| ResNet50 | / | 0.5953 | 0.9855 | **0** | 0.9974 | 1 | **0.0001** | **0** | **0.004** |
| CapsuleNet | 0.5953 | / | 0.1188 | **0** | 0.956 | 0.479 | **0** | **0** | 0.3544 |
| DBMA | 0.9855 | 0.1188 | / | **0** | 0.7211 | 0.9962 | **0.0026** | **0.0005** | **0.0002** |
| HybridSN | **0** | **0** | **0** | / | **0** | **0** | 0.8345 | 0.9862 | **0** |
| HS-CNN | 0.9974 | 0.956 | 0.7211 | **0** | / | 0.989 | **0** | **0** | **0.0301** |
| LMFN | 1 | 0.479 | 0.9962 | **0** | 0.989 | / | **0.0003** | **0** | **0.0024** |
| SPRN | **0.0001** | **0** | **0.0026** | 0.8345 | **0** | **0.0003** | / | 0.9997 | **0** |
| HPDM-SPRN | **0** | **0** | **0.0005** | 0.9862 | **0** | **0** | 0.9997 | / | **0** |
| DBSACaps | **0.004** | 0.3544 | **0.0002** | **0** | **0.0301** | **0.0024** | **0** | **0** | / |

We have added an Analysis of Variance (ANOVA) to our results section, specifically in the comparative analysis presented in Tables 4 and 5 of Section 3.1. This analysis was conducted to evaluate the performance of our model in terms of Average Accuracy, Overall Accuracy, Average Precision, Average Recall, and Average F1 Score. The ANOVA was performed under the assumption that there was no significant difference in the performance of the models, with an alpha level of significance set at 0.05. The results, as detailed in Table R1, indicate a PR value significantly less than 0.05, which effectively rejects the null hypothesis and suggests a significant difference in performance among the models.

Following the ANOVA, we conducted a post hoc analysis using the Tukey Honest Significant Difference (Tukey HSD) test to compare the models two by two. This analysis was aimed at identifying which models had significantly different performance levels, with a P-value of less than 0.05 considered indicative of significant differences. The results of the Tukey HSD analysis are presented in Table R2. This analysis revealed that our proposed model, DBSACDaps, along with HYHybirSN, SPRN, and HPDM-SPRN, showed distinct performance differences when compared to other networks. Notably, HYHybirSN, SPRN, and HPDM-SPRN exhibited overall lower values, none exceeding 0.956, while our proposed DBSACDaps model achieved overall higher values, all above 0.97.
